# Single Center Characterization of a Cohort of Salivary Gland Carcinomas

**DOI:** 10.3390/life14091089

**Published:** 2024-08-29

**Authors:** Ria Winkelmann, Maja Weißgerber, Peter J. Wild, Julia Bein, Maximilian Fleischmann, Melanie Demes, Panagiotis Balermpas, Andreas Loth, Katrin Bankov, Jens von der Grün

**Affiliations:** 1 Dr. Senckenberg Institute of Pathology, Goethe University Frankfurt, Theodor-Stern-Kai 7, 60596 Frankfurt am Main, Germany; maja.white@web.de (M.W.); peter.wild@ukffm.de (P.J.W.); julia.bein@ukffm.de (J.B.); melanie.demes@unimedizin-ffm.de (M.D.); katrin.bankov@charite.de (K.B.); 2 Frankfurt Institute for Advanced Studies (FIAS), Ruth-Moufang-Straße 1, 60438 Frankfurt am Main, Germany; 3 Department of Radiation Oncology, Goethe University Frankfurt, Theodor-Stern-Kai 7, 60596 Frankfurt, Germany; fleischmann@med.uni-frankfurt.de (M.F.); panagiotis.balermpas@usz.ch (P.B.); jens.vondergruen@usz.ch (J.v.d.G.); 4 Department of Radiation Oncology, University Hospital Zürich, Rämistrasse 100, 8091 Zürich, Switzerland; 5 Department of Otorhinolarygology, Goethe University Frankfurt am Main, Theodor-Stern-Kai 7, 60596 Frankfurt am Main, Germany; andreas.loth@unimedizin-ffm.de; 6 Department of Pediatric Oncology and Hematology, Charité-Universitätsmedizin Berlin, Freie Universität Berlin, Humboldt-Universität zu Berlin, Augustenburger Platz 1, 13353 Berlin, Germany

**Keywords:** salivary gland neoplasms, survival, MSI, immunohistochemistry, p53, Bethesda panel, IdyllaTM MSI test

## Abstract

Salivary gland cancer (SGC) is a rare cancer that can present a diagnostic challenge to pathologists, with emerging, but still limited options for the treatment of recurrent/metastatic disease. We aimed to characterize the cohort of salivary gland cancers in our institute and generate a tissue microarray (TMA) with clinical data available for immunohistochemical analysis. We extracted the cases of salivary gland cancers in our institute and generated a TMA with 72 patients between 2002 and 2017 with sufficient paraffin block material. Follow-up data were present for all cases. The TMA was stained with three p53 antibodies as well as MSH2, MSH6, PMS2 and MLH1 antibodies. Additionally, we applied fragment analysis based on the Bethesda panel, and the IdyllaTM MSI test to cases with expression loss of any of the mismatch repair proteins (MMR-P) according to our immunohistochemistry (IHC). The investigated cohort shows that pT and pN stage are the only factors independently associated with survival, according to our multivariate analysis (*p* = 0.037 and *p* = 0.014). In univariate analysis, risk factors identified in our cohort were also age (*p* = 0.015), (lympho-) vascular invasion (*p* = 0.002 and *p* = 0.003) and risk stratification (*p* = 0.037). The p53 protein investigated by three antibodies showed no statistically significant association with survival or other tumor characteristics in the investigated cohort. According to MMR-P IHC, six cases of SGC showed an aberrant IHC phenotype. Additional IdyllaTM MSI test and fragment length analysis failed to confirm microsatellite instability. The pT and pN stage are the most important factors for survival in our cohort. In our cohort, antibodies directed against the protein p53 did not contribute to clinical decision-making and were not correlated with any known clinical characteristics. MSI appears to be insignificant in SGCs. Larger cohorts are needed for verification.

## 1. Introduction

Salivary gland cancers (SGCs) are rare tumors that encompass at least 20 different tumor entities. This can sometimes pose diagnostic challenges for pathologists. According to current ASCO and WHO guidelines, salivary gland tumors are classified as either low risk or high risk [1,2]. Due to the rarity of these tumors, it is necessary to have well-characterized cohorts with follow-up data. 

Previous studies have suggested that p53 immunoreactivity patterns may serve as a marker of malignancy in these tumors [3]. Therefore, we performed p53 immunohistochemistry (IHC) to estimate p53 mutational status, which may be associated with high-grade tumor morphology and a worse overall prognosis with the goal to aid in diagnostic challenges for pathologists. This association has been observed in both mouse models and human tumors [4,5,6]. To assess the conformational status via immunohistochemistry, we aimed to use additional p53 antibody clones for staining.

Surgery and radiotherapy currently represent widely applied treatment options for these neoplasia [7]. To broaden the therapeutic toolbox, molecular marker studies have been conducted to provide options for personalized treatment regimens that have been incorporated into current ASCO guidelines [1]. Targeted therapies for SGC remain limited in the recurrent/metastatic disease setting. There is also lack of high evidence to support the use of immune checkpoint inhibition in these cancers. Trials have been conducted in a limited number of SGCs [8,9]. Low frequencies of microsatellite instability have been reported in the literature (reviewed in [10]). Therefore, another aim of this investigation was to evaluate the role of microsatellite instability (MSI) in SGC as a potential biomarker for increased sensitivity to immunotherapy, in our well-characterized cohort.

The definition of MSI subtype in several cancers is a prognostic indicator and patients may benefit from immunotherapy [11,12]. According to the European Society for Medical Oncology, two main methods are recommended for the diagnosis of MSI: IHC and fragment analysis [13]. We aimed for IHC for mismatch repair proteins (MMR-P), the IdyllaTM MSI test and fragment analysis based on the Bethesda panel.

The relationship between MSI status and prognosis has been demonstrated in several studies for different tumor types (reviewed in [14]). Immunohistochemistry was used for pre-screening and the PCR-based methods were used for validation. The rationale for using the Bethesda panel and the IdyllaTM MSI test was to investigate mono-, di- and tetranucleotide repeats as opposed to mononucleotide repeats alone, as there is currently no gold standard in this tumor entity. 

## 2. Materials and Methods

### 2.1. Patient Selection 

The archive of the Dr. Senckenberg Institute of Pathology, Frankfurt am Main was retrospectively screened for paraffin block material from surgical cases of malignant salivary gland cancer between 2002 and 2017 (*n* = 125). Cases of SGC with exceeding paraffin (FFPE) block material (*n* = 72) were retrieved from the biobank of the Dr. Senckenberg Institute of Pathology after pathological review of newly obtained hematoxylin and eosin (HE) whole-slide images presenting the current biomaterial histology. Tumor classification was performed according to the WHO classification system, 5th Edition [2]. Clinicopathological characteristics are shown in Table 1. Tumor specimens were included in the study regardless of their clinical outcome, provided that they yielded sufficient paraffin block material. Low-grade tumors were treated with function-preserving surgery. In cases where radiological examination indicated the possibility of lymph node metastasis, (selective) neck dissection was performed. In the event of pathological proven nodal metastasis, the tumor board considered the potential benefits of additional radiotherapy or heavy ion therapy. In cases where high-grade morphology was present, a neck dissection was performed following function-preserving surgery. The occurrence of pathologically proven nodal metastasis led to the possibility of a contralateral neck dissection. Cases exhibiting pathologically proven nodal metastasis or incomplete resection, in addition to tumor stages pT3 and pT4, were subject to a tumor board and further radio(chemo-)therapy or heavy ion therapy. Cases that were deemed inoperable were presented to the tumor board, where decisions were made on a case-by-case basis. In cases where palliative care was indicated, the molecular tumor board was consulted. The administered dosage to the tumor and the lymphatic drainage of radiotherapy ranged from 11 Gy to 72 Gy. In cases where palliative care was indicated or prior therapy had to be discontinued due to adverse effects, low doses were administered. 

FFPE material from resection specimens and biopsy probes was processed following diagnostic procedures. The tissue and pseudonymized patient data used in this study were provided by the University Cancer Centre Frankfurt (UCT). Written informed consent was obtained from all patients, and the retrospective study was approved by the Ethics Committee of the Medical Faculty of the University of Frankfurt (Ethikkommission des Fachbereichs Medizin der Goethe-Universität) in accordance with the Declaration of Helsinki in its current version (project number: 30-17). 

### 2.2. Preparation of a Tissue Microarray (TMA)

Current HE-stained slides were pathologically reviewed to determine disease-representative areas, including carcinoma and adjacent benign physiological tissue. Slides were digitized using a 20× brightfield slide scanner (Pannoramic Scan II, 3D Histech, Budapest, Hungary). The annotation of the defined regions of interest was set to a core diameter of 1 mm. The TMA was generated using the TMA Grand Master (3DHistech, Budapest, Hungary) and matching settings of 1 mm core diameter. The slide overlay function was instrumentalized so that digital annotations were matched to the donor tissue and transferred to an empty recipient paraffin matrix. The resulting TMA included areas of the tumor and tumor adjacent tissue to account for tumor heterogeneity. Samples of each tumor were obtained in duplicate. As negative controls, the TMA included samples of tumor-adjacent salivary gland tissue. 

### 2.3. Immunohistochemistry

TMA sections were stained with p53, MLH1, MSH2, PMS2 and MSH6 antibodies. Staining was performed using the DAKO FLEX-Envision kit (Agilent, Santa Clara, CA, USA) and the fully automated DAKO Omnis staining system (Agilent, Santa Clara, CA, USA) was applied according to the manufacturer’s instructions. The following antibodies were used: Anti-human p53 protein (DO-7, ready-to-use dilution, Dako, Agilent, Santa Clara, CA, USA), which was applied for 20 min after epitope retrieval at pH9. This antibody is routinely used in the author’s laboratory and was validated. p53 was additionally stained with clone240 (#sc-99 1:50 dilution, Santa Cruz, Santa Cruz Biotechnology, Inc., Dallas, TX, USA) 30 min after epitope retrieval at pH9. A linker as secondary antibody was added (anti-mouse, #GV821, DAKO, Agilent, Santa Clara, CA, USA). In addition, p53 staining was performed with clone 1620 (MABE339 1:25 dilution, Merck, Darmstadt, Germany): antigen retrieval for 30 min, pH9. A linker was also added (anti-mouse, #GV821, DAKO, Agilent, Santa Clara, CA, USA). Prior to utilization in the TMA, the aforementioned p53 antibodies were evaluated in the authors’ laboratory using tonsil tissue as the test material. Anti-MSH2 (G219-1129, ready-to-use dilution, Bio SB, Santa Barbara, CA, USA) applied for 60 min after epitope retrieval at pH9. Anti-MSH6 (SP93, ready-to-use dilution, DCS Inn. Diagnostik-Systeme, Hamburg, Germany) was applied for 30 min after epitope retrieval at pH9. Anti-MLH1 (BS29, ready-to-use dilution, Master Diagnóstica, Sao Paulo, Brazil) was applied for 30 min after epitope retrieval at pH9. Anti-PMS2 (EP51, ready-to-use solution, Bio SB, Santa Barbara, CA, USA) was applied for 60 min after epitope retrieval at pH9. The antibodies are employed routinely in the author’s laboratory and underwent a validation process prior to their initial use.

Epitope visualization was performed using the DAKO EnVision™ FLEX DAB+ substrate chromogen system (Agilent, Santa Clara, CA, USA). Nuclear counterstaining was performed using a DAKO hematoxylin solution (Agilent, Santa Clara, CA, USA). The slides were finally digitized.

### 2.4. Evaluation of Staining

For p53 (DO-7), staining was scored semi quantitatively: 1 for negative, 2 for staining in most tumor cells (>80%), 3 for wild-type pattern and 4 was not evaluable due to missing core on the TMA. The result was dichotomized: 1 and 2 for mutated and 3 for wild-type according to the patterns reported in the literature [15]. 

The conformation specific antibodies Pab1620 and Pab240 were evaluated as follows: definition as wild-type p53: Pab1620 positive and Pab240 negative. Mutant conformation was estimated in the case of Pab1620 negativity and Pab240 positivity as described previously [16]. 

For MSI the TMA cores were scored manually by a pathologist. In the case of more than 10% positive stained nuclei for the antibodies staining MSH2, MSH6, MLH1 and PMS2, the staining was marked as positive. Cases with no staining or less than 10% positive stained nuclei were re-evaluated using a freshly sectioned whole slide of the questionable tumor. In case of confirmation, the cases were further investigated using the IdyllaTM MSI test (Biocartis, Mechelen, Belgium) and the standard polymerase chain reaction (PCR) method (Bethesda panel). The cut-off used for MSI staining was previously described in the literature [17,18]. Each stain was checked against an internal positive control.

### 2.5. Nucleic Acid Extraction

DNA was prepared from three serial 5 µm thick and HE-stained paraffin sections. Macrodissected material was manually isolated from deparaffinized samples using the QIAamp DNA Micro Tissue Kit (Qiagen, Hilden, Germany).

### 2.6. DNA Quality Control

For quality control, DNA was analyzed using the DNA Screen Tape Assay (loading volume 2 µL) on the Agilent 4200 TapeStation (Agilent Technologies, Santa Clara, CA, USA). This automated electrophoresis solution is also used to determine the integrity. The DNA Integrity Number (DIN) is a software algorithm for determining DNA quality. DIN values range from 1 to 10, with 10 being completely intact DNA and 1 being completely degraded.

### 2.7. MSI Testing by Fragment Analysis

Microsatellite analysis was performed by a fluorescent multiplex PCR-based method and fragment analysis using the ABI 3010xl Genetic Analyzer (Applied Biosystems, Foster City, CA, USA). The allelic profiles of microsatellite markers generated by amplification of matched tumor and normal tissues were compared. Panel 1 (BAT25, BAT26, D2S123, D5S346 and D17S250) and panel 2 (BAT40, D10S197, MYCl, NR21 and NR24) contain two different analyses of five microsatellite systems each. Therefore, a total of 10 microsatellite markers were used for MSI testing.

### 2.8. IdyllaTM MSI Assay

For the IdyllaTM MSI assay 1 × 10 µm FFPE tissue was freshly sectioned after pathological assessment of tumor cell content. A PCR reaction was applied following a melting curve analysis with the markers ACVR2A, BTBD7, DIDO1, MRE11, RYR3, SEC31A and SULF2 [19]. These tumor-specific biomarkers do not require the analysis of paired normal tissue samples as is the case with the fluorescent multiplex PCR-based method. No upstream DNA extraction is required. A neoplastic cell content of at least 20% is required. If at least two mutant biomarkers are present, the system flags the case as MSI. None or one mutated biomarker indicates MSS phenotype by this system [20]. 

Output examples for fragment analysis based on the Bethesda panel and the IdyllaTM MSI test are shown in Supplementary Figure S1. 

### 2.9. Statistical Analysis

Tests for normal distribution of the data were performed using the Kolmogorov–Smirnov test and the Shapiro–Wilk test using IBM^®^ SPSS^®^ Statistics 26 (IBM Corp., IBM SPSS Statistics for Windows, Armonk, NY, USA). No normal distribution was found, so additional tests were required: Spearman’s rank correlation as well as chi-squared test and Fisher’s exact test. In addition, the log rand test for Kaplan–Meier curves and Cox regression analysis was applied. Bonferroni correction was applied and marked. Statistical significance was considered at *p* < 0.05.

## 3. Results

### 3.1. Characteristics of the Investigated Cohort

**Figure 1:**
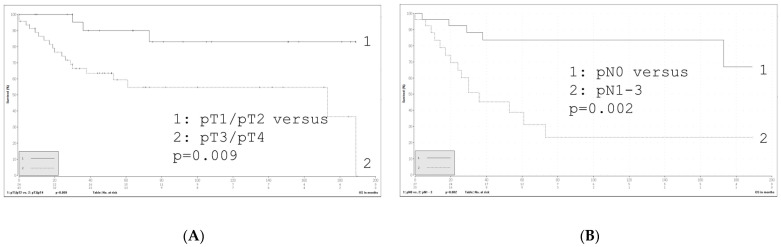
Kaplan–Meier curves for the investigated cohort regarding overall survival in months with regards to pT (**A**) and pN (**B**).

Our cohort of 72 malignant salivary gland tumors was dichotomized into the groups low-risk and high-risk according to current standard [21]. Adenoid cystic carcinomas were assigned to the high-risk group. Characteristics are presented in Table 2 stratified by risk group. The median age in this group is 61 years (range 17–91). According to the Chi square test, a statistically significant relationship between the variables low risk and high risk and sex (*p* = 0.002), pathologically assessed nodal stage (pN) (*p* < 0.001), as well as lymphatic vessel invasion (L) (*p* < 0.001) and perineural invasion (Pn) (*p* = 0.002) was detected. No statistically significant relationships were detected for age, tumor localization, tumor stage (pT), distant metastasis (cM), vessel invasion (V) and p53 (clone DO-7) and MSI IHC via the Chi square test. Cox regression analysis shows a statistically significant association for the parameters age, pT stage, pN, (lymphatic) vessel invasion (L and V) and risk groups in the univariate analysis regarding overall survival. An additional multivariate analysis left pT and pN stage as the only significant factors (see Table 3). The Kaplan–Meier estimator is presented in Figure 1. 

### 3.2. p53 Status in Relation to Tumor Characteristics

In the tumor tissue TMA 37/72 cases (51%) showed a wild-type pattern and 35/72 (49%) showed an aberrant pattern (either complete loss or strong expression). p53 data were dichotomized and correlated with known tumor characteristics. There was a statistically significant correlation between p53 staining and MSI status by immunohistochemistry (*p* = 0.013 prior to Bonferroni correction) as calculated by Chi square test. There was no significant association between p53 (clone DO-7) status and other tumor characteristics. Data are presented in Supplementary Table S1A. Cox regression analysis showed no significant association between overall survival and p53 (clone DO-7) data in the cohort of malignant salivary gland neoplasms (*p* > 0.05, Table 3). 

Further staining was performed using the clones Pab1620 and Pab240 for conformational analysis of p53. Two cases showed a staining pattern suggestive of mutant conformation: A case of carcinoma ex pleomorphic adenoma and one case of salivary duct carcinoma. With the clone DO-7, the salivary duct carcinoma showed wild-type staining pattern and the carcinoma ex pleomorphic adenoma showed a mutant specific pattern using the clone DO-7. All these cases (*n* = 2) showed an MSS phenotype by immunohistochemistry.

### 3.3. MSI Status in Relation to Immunohistochemistry

**Figure 2:**
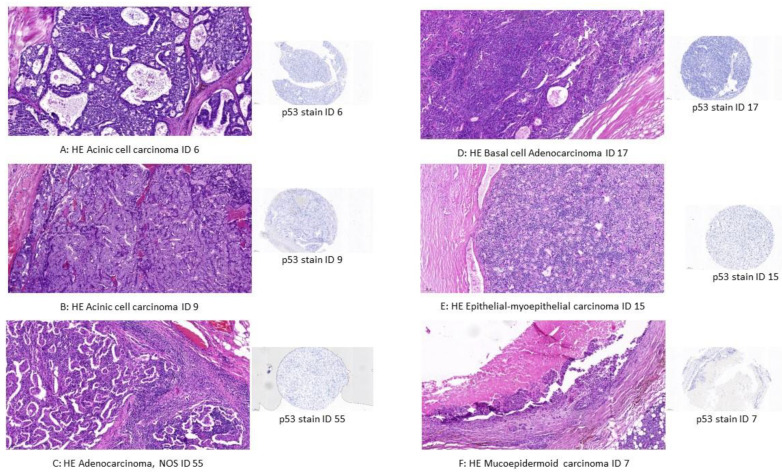
HE-stained slides of salivary gland carcinomas with immunohistochemical loss of expression for mismatch repair proteins (MLH1, PMS2, MLH6, MSH2) in the TMA setting; magnification 10-fold; scale: 100 µm with p53 TMA cores. (**A**) Acinic cell carcinoma (case 6); p53 TMA core. (**B**) Acinic cell carcinoma (case 9); p53 TMA core. (**C**) Adenocarcinoma, NOS (case 55); p53 TMA core. (**D**) Basal cell adenocarcinoma (case 17); p53 TMA core. (**E**) Epithelial-myoepithelial carcinoma (case 15); p53 TMA core. (**F**) Mucoepidermoid carcinoma (ID 7); p53 TMA core.

We stained the TMA sections for the antibodies MLH1, MSH6, PMS2 and MSH2. In total, 68 TMA cores were evaluable. Four cores were not present on the slide. In the tumor cohort (*n* = 68), eight cases (8/68, 12%) showed no staining for one or more markers on the TMA sections. To confirm the staining results, we evaluated freshly sectioned wholemount slides of the tumors that received a negative score. The staining results were confirmed in most cases (6/8, 75%). These cases consisted of two acinic cell carcinomas (ACCs) (2/6, 33%), one adenocarcinoma, NOS, one basal cell adenocarcinoma (BAC), one epithelial-myoepithelial carcinoma and one mucoepidermoid carcinoma (MEC) (each 1/6, 17%). The cases are displayed in Figure 2, which also includes p53 staining (clone DO-7). Apart from the beforementioned correlation between p53 staining and MSI status by immunohistochemistry, there was no significant association between MSI IHC status and other tumor characteristics. Data are presented in Supplementary Table S1B. Cox regression analysis showed no significant association between overall survival and MSI IHC data in the cohort of malignant salivary gland neoplasms (*p* > 0.05, Table 3). 

### 3.4. MSI Status According to IdyllaTM MSI Test

Six cases with staining results suspicious for MSI were subjected to the additional IdyllaTM MSI testing after confirmation using wholemount slides. A valid test result was obtained in 4/6 (67%) cases. All evaluable cases (*n* = 4) were classified as MSS according to the IdyllaTM MSI test. The cases classified as invalid showed failure in all genes tested (ID 17) in one case and failure in the genes BTBD7, MRE11, RYR3, SULF2 in the other case (ID 7). The results are presented in Table 4.

### 3.5. DNA Quality Control and MSI Status by Fragment Analysis

DNA concentrations and DIN numbers are shown in Supplementary Table S2. DNA concentrations ranged from ca. 0.7 ng/µL to 51 ng/µL. DIN ranged from 1.6 to 3.2. In one case the sample concentration was outside the functional range for DIN and the assay. 

Fragment analysis was performed on the two Bethesda panels. A result was obtained in two out of six cases (33%). No case was classified as MSI. No valid result was obtained in two cases (2/6, 33%). Two cases (2/6, 33%) showed a result classified as proficient in at least one panel (see Table 4). 

## 4. Discussion

Salivary gland carcinomas are uncommon tumors that present a variety of diagnostic challenges for pathologists. We extracted salivary gland carcinomas from the archive of the Dr. Senckenberg Institute of Pathology to create a TMA. Clinical and follow-up data as well as data about clinical treatment were available for our cases. Collectives of salivary gland carcinomas are important due to the rarity and heterogeneity of these tumors. Therefore, a cohort of patients with known clinical data is presented here that allows studying these tumors. 

Grading and tumor stage are by now the most reliable prognostic factors, and surgery remains the preferred treatment strategy for patients with SGC in most cases [7]. Our cohort shows that the pT and pN stage is the only factor associated with survival, according to our multivariate analysis. In univariate analysis, risk factors identified in our cohort were also age, (lympho-) vascular invasion and risk stratification. 

TMAs allow for simultaneous immunohistochemical analysis of a sample cohort under the same conditions. It is well-established that over 50% of tumors exhibit a p53 mutation, and that p53 can be inactivated in many other types of cancer [22]. Therefore, we sought to investigate the role of p53 in our salivary gland cancer cohort. We stained the TMA with an antibody against the protein p53. p53 is a commonly used marker in routine diagnostics to aid in tumor diagnosis when there is overexpression or complete loss. In response to oncogene activation, DNA damage and other stress signals, it can induce cell cycle arrest, senescence and apoptosis [23]. A study from Finland found no association between p53 IHC and survival in SGC [24], which is in line with our observations. Another study indicated that TP53 mutations are not a common occurrence in salivary gland neoplasms and that p53 immunopositivity is not linked to sequence mutations [25]. We did not sequence the tumors for p53, but to depict this problem, we performed staining with two additional antibodies (Pab240 and Pab1620). We found two cases suggestive for mutation specific staining pattern. There was a discrepancy between the patterns between DO-7 and Pab240 and Pab1620. This difference may have various reasons owing to the plasticity of this gene and mutations that are not detected by the antibodies [26]. Therefore, to further elucidate the role of p53 in SGC, a sequencing approach should be aspired to, which was not performed because of poor DNA quality. 

We additionally tested the tumors for MSI status using IHC and the Bethesda panel as well as the IdyllaTM MSI test. The Bethesda panel and the IdyllaTM MSI test were applied to cases that showed a loss of expression of MSI markers after whole-slide staining according to the commonly used algorithm in routine colorectal cancer specimens [27,28]. In our cohort no MSI-positive SGCs could be detected using the IdyllaTM MSI based test (mononucleotides) or the Bethesda panel for the valid cases. 

The question needs to be raised whether this IHC combination is an adequate test method for the determining MSI status in salivary gland neoplasms. 

The MMR system comprises seven protein markers: hMLH1, hMLH3, hMSH2, hMSH3, hMSH6, hPMS1 and hPMS2 [29]. Further investigations are required for the remaining proteins in salivary gland neoplasms. 

It is known that in colorectal cancer, the correlation between immunohistochemical microsatellite status and Lynch syndrome is almost 100% [30]. Other tumor entities show different results when IHC and PCR-based methods are examined in parallel, i.e., for breast and brain tumors an association of only 35% has been described [31]. However, there was no association between IHC and PCR-based methods in this study. A possible reason for this may be the fact that the IdyllaTM MSI test has an IVD-CE mark for colorectal adenocarcinoma tissue samples but not for other tumors and is focused on mononucleotides only. Therefore, validation is required for testing in other tumors. In addition, the IdyllaTM MSI test defines MSI-L as MSS, which may not always be true for other tumors such as endometrial carcinomas [32]. Furthermore, as previously shown, samples with poor DNA quality or low tumor cellularity can lead to false negative results [32]. The samples examined in this study showed impaired DNA quality and degradation, which could be a reason for false negative results. 

A pitfall of fragment analysis using the Bethesda panel, which is established for colorectal carcinomas, is the complex MSI profiles in other tumors such as endometrial carcinomas. A small overall reading shift may lead to misinterpretation of the data [32]. Studies using the Bethesda panel require paired non-neoplastic tissue, which limits its use in routine practice. Other PCR test systems used include Promega^®^ panel, Diatech Pharmacogenetics Titiano MSI and Biotype/Modaplex, as reviewed in [33]. The Bethesda panel is a panel that interrogates mono-, di- and one tetranucleotide repeat, whereas the IdyllaTM MSI test only interrogates mononucleotide repeats, which is a limitation of this test system. Moreover, hypermethylation in the promoter regions of hMLH1 and hMSH2 can be associated with MSI status, as described in head and neck cancers [34]. Therefore, additional research is needed. 

Other studies investigating the role of MSI in SGC found no association between MSI and SGC cancer (0/34) [35] or an association in 20% of the cases (2/10) [36]. Both studies also investigated p53 status in SGC. In conclusion, both studies suggest a role for p53 expression in SGC, which cannot be found in the present study. 

A limitation of this study is that the number of cases and corresponding histologic subgroups is insufficient to yield statistically valid results. Another limitation of the study is that the IdyllaTM MSI test and the Bethesda panel assay were not applied to the IHC-MSS samples so that the false negative rate in this cohort cannot be determined. 

IHC is easy to assess and interpret in most cases of strong nuclear staining. However, impaired fixing times can lead to faint staining and reduced immunoreactivity, making evaluation more difficult. Despite this, IHC is an approved surrogate screening method for MSI [32]. 

The IdyllaTM MSI test has a faster turnaround time compared to fragment analysis, which requires more hands-on and lab time, due to the need for nucleic acid extraction and analysis of matching normal and neoplastic tissue. Furthermore, experience is required for the evaluation of fragment analysis results, whereas the readout of the IdyllaTM MSI test is clearly stated in the report (see Supplementary Figure S1). 

The techniques applied here are modules that can be implemented in larger NGS panels to detect common genetic alterations in SGC [37]. This technique is more time- and cost-consuming in routine use [33]. 

## 5. Conclusions

Creating a TMA for this rare tumor entity provides a rapid and efficient method to test immunohistochemical markers on a large, well-defined and heterogeneous group of salivary gland cancers.

In our cohort, there was no statistically significant association between tumor characteristics and p53 protein staining. Further investigations are needed to sequence and detect (epi-)genetic changes of p53 in SGCs, to rule out staining artefacts. 

Based on PCR results, MSI appears to be insignificant in SGCs. Larger cohorts are needed for verification. 

Further research is warranted to validate our findings and explore novel therapeutic approaches for this rare but clinically significant malignancy.

## Figures and Tables

**Table 1:** Characteristics of the investigated cohort (*n* = 72).

Characteristics		*n*	%
Gender	Male	43	60
	Female	29	40
Localization	Parotid gland	53	74
	Submandibular gland	6	8
	Minor salivary glands	13	18
pT	1	20	28
	2	5	7
	3	37	51
	4	10	14
pN	0	29	40
	pN1 or higher	27	38
	X	16	22
L	0	44	61
	1	28	39
V	0	65	90
	1	7	10
Pn	0	50	69
	1	21	29
	X	1	1
R	0	44	61
	1	20	28
	2	1	1
	X	7	10
cM	0	66	92
	1	3	4
	X	3	4
Histology	Acinic cell carcinoma	9	13
	Adenocarcinoma (NOS)	15	21
	Adenoid cystic carcinoma	10	14
	Basal cell adenocarcinoma	2	3
	Carcinoma ex pleomorphic adenoma	6	8
	Epithelial-myoepithelial carcinoma	2	3
	Mucoepidermoid carcinoma	15	21
	Myoepithelial carcinoma	1	1
	Polymorphous adenocarcinoma	3	4
	Salivary duct carcinoma	9	13

Abbreviations: pT: pathologically assessed tumor stage; pN: pathologically assessed nodal stage; L: lymphovascular invasion; V: vascular invasion; Pn: perineural invasion; R: resection margin status; cM: clinically assessed metastatic status.

**Table 2:** Characteristics of investigated cases stratified by risk.

		Low Risk (*n* = 27)		High Risk (*n* = 45)		
		n	%	n	%	p (Χ2)
Age	<61	15	56	23	51	0.715
	≥61	12	44	22	49	
Sex	Male	10	37	33	73	0.002
	Female	17	63	12	27	
Localization	Parotid	19	70	34	76	0.265
	Submandibular gland	1	4	5	11	
	Glandulae minores	7	26	6	13	
pT	1	11	41	9	20	0.129
	2	3	11	2	4	
	3	10	37	27	60	
	4	3	11	7	16	
pN	0	17	63	12	27	<0.001
	pN1–3	0	0	27	60	
	X	10	37	6	13	
cM	0	25	93	41	91	0.182
	1	0	0	3	7	
	Missing	2	7	1	2	
L	0	26	96	18	40	<0.001
	1	1	4	27	60	
V	0	26	96	39	87	0.182
	1	1	4	6	13	
Pn	0	25	93	25	56	0.002
	1	2	7	19	42	
	X	0	0	1	2	
p53 IHC ^1^	WT	14	52	23	51	0.951
	Mut	13	48	22	49	
MSI IHC	Proficient	23	85	39	87	0.158
	Deficient	4	15	2	4	
	NA	0	0	4	9	

^1^ p53 according to DAKO clone DO7.

**Table 3:** Univariate (A) and multivariate (B) Cox regression analysis.

		A Univariate Cox Regression Analysis				B Multivariate Cox Regression Analysis			
Characteristics	Categorization	*p*	Hazard Ratio	Confidence Interval		*p*	Hazard Ratio	Confidence Interval	
Age	<61 years/≥61 years	0.015	0.337	0.14	0.809	0.122	0.476	0.186	1.218
Gender	Male/female	0.636	0.81	0.338	1.94				
Localization	Parotid/other localizations	0.058	0.467	0.213	1.026				
pT	pT1, pT2/pT3, pT4	0.009	5.111	1.497	17.45	0.037	3.943	1.089	14.273
pN	pN0/pN > 0 excl. pNX	0.002	5.131	1.839	14.312	0.014	25.387	1.928	334.365
cM	M0/M1	0.443	0.039	0.000	153.661				
L	L0/L1	0.002	4.124	1.708	9.961	0.085	2.677	0.873	8.205
V	V0/V1	0.003	5.07	1.742	14.757	0.296	1.853	0.583	5.883
Pn	Pn0/Pn1	0.562	1.082	0.829	1.411				
Risk factor	Low risk/high risk	0.037	0.315	0.106	0.934	0.455	0.610	0.166	2.235
p53 IHC (DO-7)	WT/Mut	0.790	1.127	0.468	2.71				
MSI IHC	MSS/MSI	0.996	0.997	0	3.643				

**Table 4:** Staining results for the MSI markers on the TMA, IdyllaTM MSI test, fragment length analysis.

ID	Morphologic Subtype	MLH1 IHC	PMS2 IHC	MSH6 IHC	MSH2 IHC	Idylla^TM^ MSI TEST	Fragment Length Analysis
6	Acinic cell carcinoma	0	NA	NA	0	MSS	1/2 MSS *
9	Acinic cell carcinoma	0	0	0	0	MSS	MSS
55	Adenocarcinoma, NOS	NA	0	0	0	MSS	1/2 MSS *
17	Basal cell adenocarcinoma	0	0	0	0	invalid	invalid
15	Epithelial-myoepithelial carcinoma	0	0	0	0	MSS	MSS
7	Mucoepidermoid carcinoma	0	0	0	0	invalid	invalid

0: negative; NA: not analyzable. MSS: microsatellite stable. * One of two systems showed the MSS phenotype.

## Data Availability

The datasets used and/or analyzed during the current study are available from the corresponding author on reasonable request.

## Supplementary Materials

The following supporting information can be downloaded at: https://www.mdpi.com/article/10.3390/life14091089/s1, Table S1: A and B IHC p53 und MSI; Table S2: DNA concentrations and DIN numbers of extracted DNA; Figure S1: MSI Methods.
