# Supplementary figures and images for: Single Center Characterization of a Cohort of Salivary Gland Carcinomas
Source: Life (Basel). 2024 Aug 29;14(9):1089. doi: 10.3390/life14091089 (PMC11432769; doi:10.3390/life14091089)

A

1

Sample 1

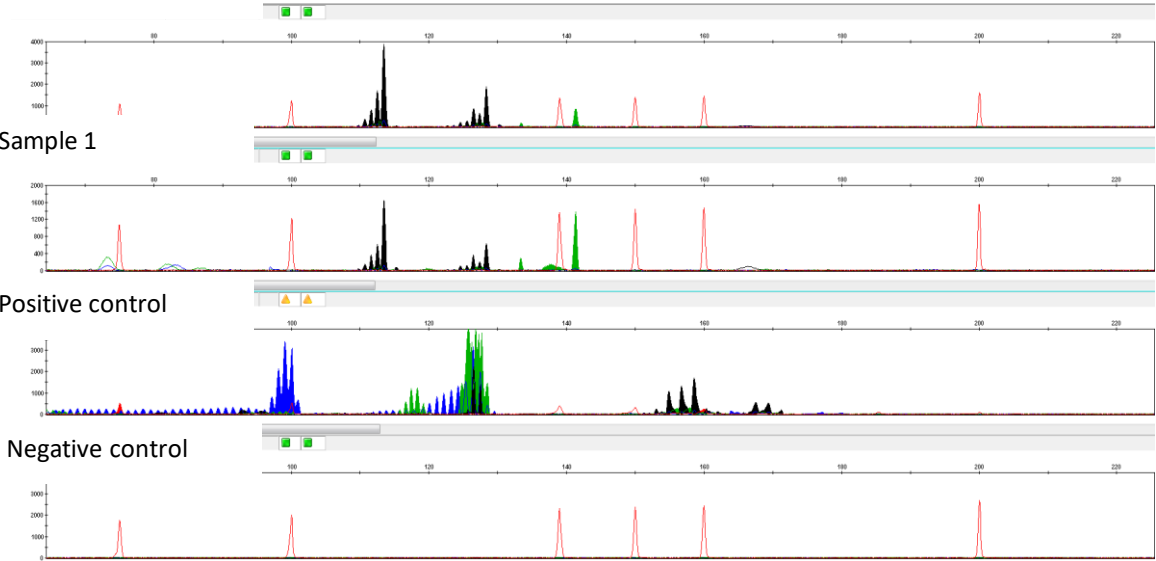

2

Sample 1

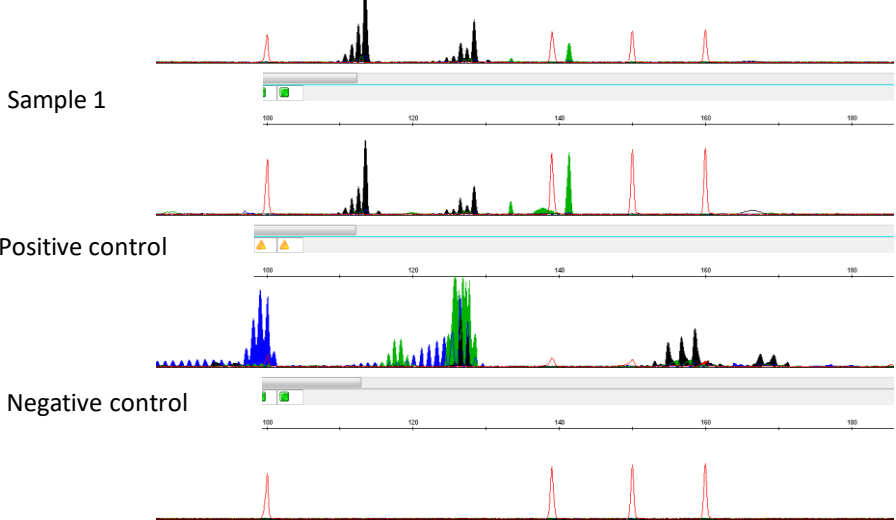

B

SEPTOPUS™ Sample 1

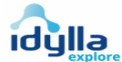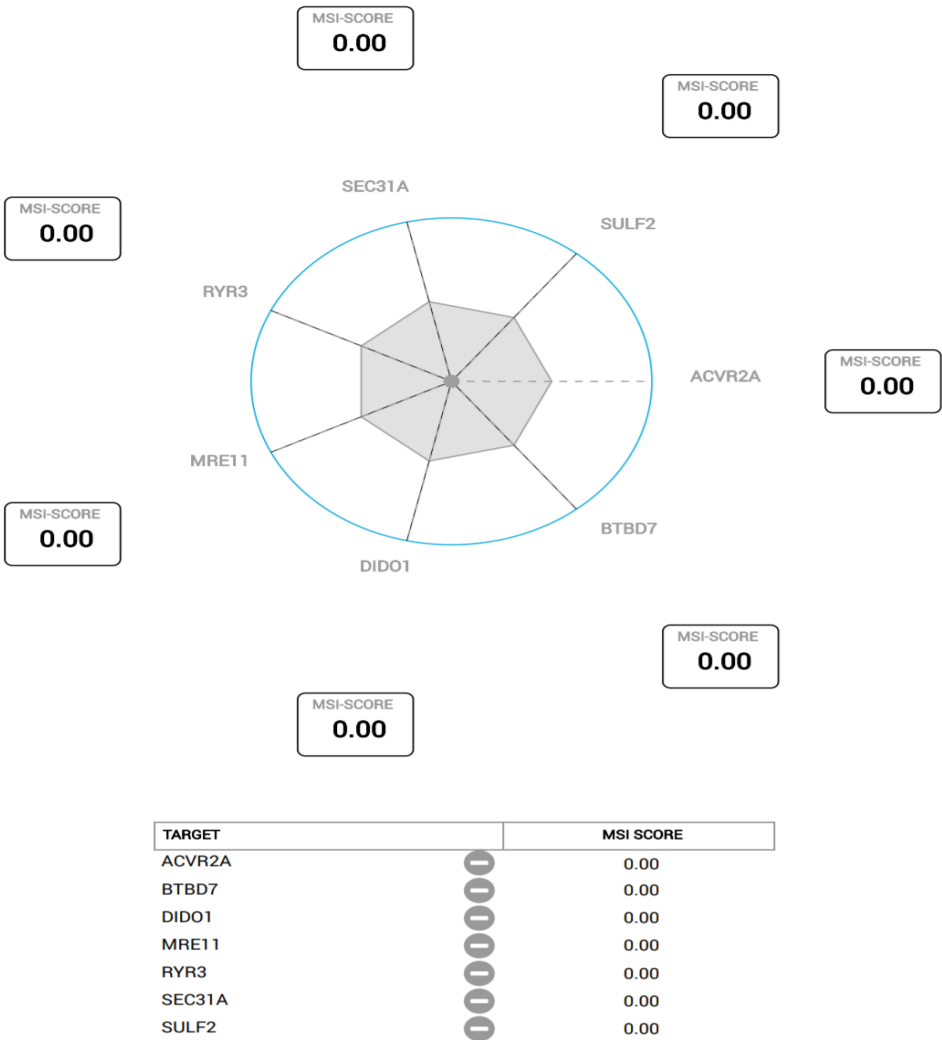

Supplementary Figure S1.

Supplement: Supplementary file 1 [file life-14-01089-s001.zip › Supplementary Figure S1_MSI Methods.pdf]
